# Overexpression of the Large-Conductance, Ca^2+^-Activated K^+^ (BK) Channel Shortens Action Potential Duration in HL-1 Cardiomyocytes

**DOI:** 10.1371/journal.pone.0130588

**Published:** 2015-06-19

**Authors:** Joseph R. Stimers, Li Song, Nancy J. Rusch, Sung W. Rhee

**Affiliations:** Department of Pharmacology & Toxicology, University of Arkansas for Medical Sciences, Little Rock, Arkansas, United States of America; Monell Chemical Senses Center, UNITED STATES

## Abstract

Long QT syndrome is characterized by a prolongation of the interval between the Q wave and the T wave on the electrocardiogram. This abnormality reflects a prolongation of the ventricular action potential caused by a number of genetic mutations or a variety of drugs. Since effective treatments are unavailable, we explored the possibility of using cardiac expression of the large-conductance, Ca^2+^-activated K^+^ (BK) channel to shorten action potential duration (APD). We hypothesized that expression of the pore-forming α subunit of human BK channels (hBKα) in HL-1 cells would shorten action potential duration in this mouse atrial cell line. Expression of hBKα had minimal effects on expression levels of other ion channels with the exception of a small but significant reduction in Kv11.1. Patch-clamped hBKα expressing HL-1 cells exhibited an outward voltage- and Ca^2+^-sensitive K^+^ current, which was inhibited by the BK channel blocker iberiotoxin (100 nM). This BK current phenotype was not detected in untransfected HL-1 cells or in HL-1 null cells sham-transfected with an empty vector. Importantly, APD in hBKα-expressing HL-1 cells averaged 14.3 ± 2.8 ms (n = 10), which represented a 53% reduction in APD compared to HL-1 null cells lacking BKα expression. APD in the latter cells averaged 31.0 ± 5.1 ms (n = 13). The shortened APD in hBKα-expressing cells was restored to normal duration by 100 nM iberiotoxin, suggesting that a repolarizing K^+^ current attributed to BK channels accounted for action potential shortening. These findings provide initial proof-of-concept that the introduction of hBKα channels into a cardiac cell line can shorten APD, and raise the possibility that gene-based interventions to increase hBKα channels in cardiac cells may hold promise as a therapeutic strategy for long QT syndrome.

## Introduction

Long QT syndrome (LQTS) is characterized by a prolongation of the ventricular action potential, resulting in an increased duration between the Q wave and the T wave on the electrocardiogram (ECG). This electrophysiological abnormality is a potentially life-threatening condition, because prolongation of the ventricular action potential (AP) can trigger lethal arrhythmias including *torsade de pointes*. Symptoms of LQTS range from mild palpitations to fainting or ventricular fibrillation and sudden death [1]. LQTS has a prevalence of 1 in 2,000 to 10,000 people and an estimated 50,000 Americans have LQTS with 3,000 deaths attributed annually to LQTS-related arrhythmias [2]. Long QT syndrome has a variety of underlying causes including genetic mutations and drug-induced abnormalities of ventricular repolarization. To date, 13 different genes with multiple mutations at each gene have been linked to LQTS [1,3]. Most of these genes encode ion channels including the hERG type K^+^ channel, Na^+^ channel, and L-type Ca^2+^ channel, but others encode various structural proteins, including caveolin 3, ankyrin and A-kinase anchoring protein 9 (AKAP) [1,3]. Mutations in the Na^+^ channel (LQT3) and L-type Ca^2+^ channel (LQT8) result in a gain-of-function to enhance depolarizing cation currents during the ventricular AP, whereas the other mutations cause a loss of function in their respective proteins [4]. There is no highly effective treatment for LQTS, although prevention of arrhythmias is attempted with β-adrenergic receptor blocker therapy, because β1-adrenergic stimulation of the heart often exacerbates arrhythmias associated with LQTS [1]. Additionally, arrhythmia termination can sometimes be accomplished with an implantable cardio-defibrillator [5]. However, these interventions are only partially effective and not curative.

### HL-1 cells, a murine atrial cell line

The rapidly activating delayed-rectifier K^+^ channel (I_Kr_; Kv11.1 or KCNH2) contributes to the K^+^ efflux that mediates repolarization in HL-1 cells [6]. These cells are a mouse cardiac cell line derived from an atrial tumor and are amenable to genetic and pharmacological manipulations [7,8]. HL-1 cells were recently demonstrated to possess I_Kr_ with properties comparable to native cardiac I_Kr_, thereby providing an experimental model suitable for studies of I_Kr_ channels [9,10]. In ventricular myocytes, including those isolated from human left ventricle [11], the voltage-dependent I_Kr_ channel generates the outward K^+^ current partially responsible for repolarization of the cardiac AP, and inhibition of I_Kr_ is a mechanism by which many drugs induce LQTS [12].

Two other prominent K^+^ channels, the transient outward K^+^ channel (I_to_; Kv4.3) and the slowly activating delayed-rectifier K^+^ channel (I_Ks_; Kv7.1), also contribute to repolarization of the AP in HL-1 cells [13]. I_to_ exerts its influence in the early phases of repolarization (phase 1 on the electrocardiogram) while I_Ks_ is active during the late phase of repolarization (phase 3). Although not as significant as I_Kr_, I_to_ and I_Ks_ can shorten AP duration (APD) in HL-1 cells [14]. In humans, mutations in I_Ks_ underlie the majority of the cases of genetic LQTS (LQT1) [1].

### Expression of BK channels to shorten APD

Here, we explored the hypothesis that introducing the α-subunit (BKα) of the large-conductance, Ca^2+^-activated K^+^ (BK) channel into HL-1 cells can shorten APD in this cardiac cell line. BK channels are not normally expressed in cardiac plasma membranes, although they have been reported in mitochondrial membranes, where they may contribute to mitochondrial K^+^ uptake in myocytes, and may protect the myocardium against infarction [15,16]. In contrast, BK channels play an important role in repolarization of neuronal action potentials [17], and are one of several key K^+^ channels that restore resting membrane potential to depolarized vascular smooth muscle cells and other excitable cell types [18]. The properties of BK channels include Ca^2+^-sensitivity, voltage-sensitivity, and a large single-channel conductance of 200–300 pS in symmetrical K^+^ solutions [16]. These properties imply that BK channels could be activated during the plateau phase of the AP to generate a powerful repolarizing current after introduction into cardiac myocytes. Finally, although the BK channel pore-forming structure represents a tetramer composed of four BKα subunits, a single gene encodes all BKα, unlike most other types of voltage-gated K^+^ channels whose pore-forming structures are co-assemblies of multiple α-subunit subtypes [19,20]. From the logistical perspective, this feature would simplify the process of expressing BK channels in cardiac cells, requiring introduction of only a single BKα gene to accomplish a well-defined channel pore. However, it does not account for the possibility that ancillary BK channel subunits or modulating proteins that confer full physiological function to the channel, may be absent in cardiac myocytes. For example, small beta subunits (BKβ) are known to enhance the Ca^2+^-sensitivity of the BK channel, enabling it to open in response to small increases in [Ca_i_], and thereby tightly titrating the level of repolarizing current [19,21]. Thus, it is unknown whether expressing BK channels in cardiac cell preparations can generate functional K^+^ current and shorten APD, a question we explored here initially in the HL-1 cell model.

## Materials and Methods

### Ethics Statement

C57BL/6J adult mice were anesthetized deeply with 2.5% isoflurane and sacrificed by decapitation before removal of brain tissue. This study was carried out in strict accordance with the recommendations in the Guide for the Care and Use of Laboratory Animals of the National Institutes of Health. The protocol was approved by the Institutional Animal Care and Use Committee of the University of Arkansas for Medical Sciences (File Number: 3290). The HL-1 cell line derived from a mouse atrial tumor was established in 1998 [7] and was provided as a gift to Dr. Stimers in 2001.

### Cell culture

HL-1 cells for the present study were cultured as previously described [7] in T25 flasks for passaging or plated in 35 mm tissue culture dishes for electrophysiology. Cells were cultured in supplemented Claycomb media (Sigma/Aldrich, St. Louis, MO) containing the following: 87% Claycomb medium, 10% fetal bovine serum, 1% penicillin/streptomycin, and 2 mM l-glutamine. HL-1 cells were passaged when they reached confluency (3–5 days) by exposure to 0.05% trypsin/EDTA solution for 10–12 min at 37°C. HL-1 cells were suspended in supplemented Claycomb media and then plated in the flasks or dishes. The cells were incubated at 37°C in an incubator with 5% CO_2_ / 95% air.

### Plasmids and transfection

A triple Flag tag (DYKDHDGDYKDHDIDYKDDDDK) was commercially synthesized and fused (GenScript, Piscataway, NJ) to the gene encoding hBKα (GenBank: U23767.1) in a vector backbone that included a bicistronic expression of mCherry for detection of transfected cells (a gift from Dr. Sarah England, Washington University). A vector containing mCherry, but lacking the hBKα gene, was used as a negative control (Null). HL-1 cells were transfected 24 hr after plating on 35 mm tissue culture dishes, when they had attained 50% to 70% confluency. Cells were transfected with 2 μg DNA per 35 mm dish using Lipofectamine LTX and Plus reagent according to manufacturer’s directions (Invitrogen, Carlsbad, CA). Cells were used in experiments 48 to 72 hr after transfection.

### Immunofluorescence labeling and detection

HL-1 cells were grown on glass coverslips, fixed and immunostained, and mounted on glass slides as previously described [22]. Anti-Flag antibody (1:2000, Sigma-Aldrich) and DyLight 488 anti-mouse antibody (1:5000, Jackson ImmunoResearch, West Grove, PA) were used as primary and secondary antibodies. Cells were imaged with an Orca ER camera (Hamamatsu, Houston, TX) and 63x NA1.4 objective on an Axiovision 200M microscope (Zeiss, Thornwood, NY). Color images were overlaid with IPLab 4.0 software (BD Biosciences, San Jose, CA).

### Western Blot

HL-1 cells were lysed using RIPA buffer (Thermo Fisher, Waltham, MA) containing protease inhibitor cocktail (Roche, South San Francisco, CA). Equal amounts of protein (10–20 μg) were loaded into wells of 7% or 3–8% tris-acetate gels, subjected to SDS-PAGE, and transferred to a 0.45 μm PVDF membrane (Thermo Fisher). Western blot analyses were performed using the following primary antibodies: anti-Flag (Sigma-Aldrich), anti-Kv4.3 (Alomone, Jerusalem, Israel), anti-Kv11.1 (Alomone), anti-Nav1.5 (Alomone), anti-Cav1.2 (Alomone), and anti-GAPDH (Millipore, Billerica, MA). The immunodensity of bands corresponding to targeted proteins were quantified using NIH ImageJ software, and values were corrected for protein loading by dividing them by GAPDH immunodensity signals in the same lanes. Subsequently, corrected values obtained from Western blots loaded with protein lysate from BKα-transfected cells were normalized to corrected values obtained from Null-transfected cells corresponding to the same ion channel. According to this analysis, by definition the average normalized intensity of Null transfection is unity. For Western blots designed to detect Kv11.1, both bands of the Kv11.1 doublet revealed by anti-Kv11.1 blotting were included in calculations.

### Patch clamp

Following transfection of HL-1 cells with either hBKα or Null plasmids for 48 to 72 hr as described above, myocytes were trypsinized (0.05% trypsin/EDTA for 10–12 min) and a drop of the cell suspension transferred to a glass bottom perfusion chamber on the stage of an inverted microscope. The chamber was perfused intermittently with control HBSS of the following composition (in mM): 145 NaCl, 5 KCl, 2 CaCl_2_, 1 MgCl_2_, 10 HEPES, and 5.5 dextrose (pH 7.4). Patch electrodes were filled with a K^+^-aspartate solution consisting of (in mM): 115 KOH, 115 DL-aspartic acid, 30 KCl, 1 EGTA, 10 HEPES, 1 MgATP and 10 nM or 300 nM free CaCl_2_ (pH 7.2). All experiments were performed at room temperature.

Electrophysiological measurements were made using conventional techniques and methods previously described [23,24]. Data obtained in response to voltage or current pulses were low pass filtered by either analog or digital filters at 1 to 5 kHz and sampled at 2 to 10 kHz as appropriate for channel kinetics. Patch pipettes were made from borosilicate glass (8250, King Precision Glass, Claremont, CA) pulled on a Brown-Flaming P77 puller (Sutter Instruments, Novato, CA) and fire polished to have a final resistance of 3–5 MΩ (inside tip diameter of 0.5–1 μm). Capacitance and series resistance compensation were used to improve the quality of the voltage clamp and reduce associated artifacts. Whole-cell currents were elicited from a holding potential of -70 mV with 500 ms pulses to potentials between -60 and +60 in 10 mV increments. P/4 protocols were used to cancel leak and capacitance currents when pulse protocols were applied. Action potentials were recorded in current-clamp mode by applying a 1 ms pulse of 5 pA at 0.5 Hz following a 100 ms pulse of -5 pA to elicit an anode break excitation.

### Statistical Analysis

All experiments were replicated 5 to 10 times. Measured or calculated parameters are reported as mean ± SEM. Currents were normalized to cell size by dividing by capacitance. All experiments were analyzed by unpaired Student's t-test, except for comparisons of I-V curve amplitudes, which were analyzed using analysis of variance with a Tukey post-hoc test. The criterion for a significant difference was a P value less than 0.05.

## Results

### Expression of hBKα and other ion channels in HL-1 Cells

**Fig 1:**
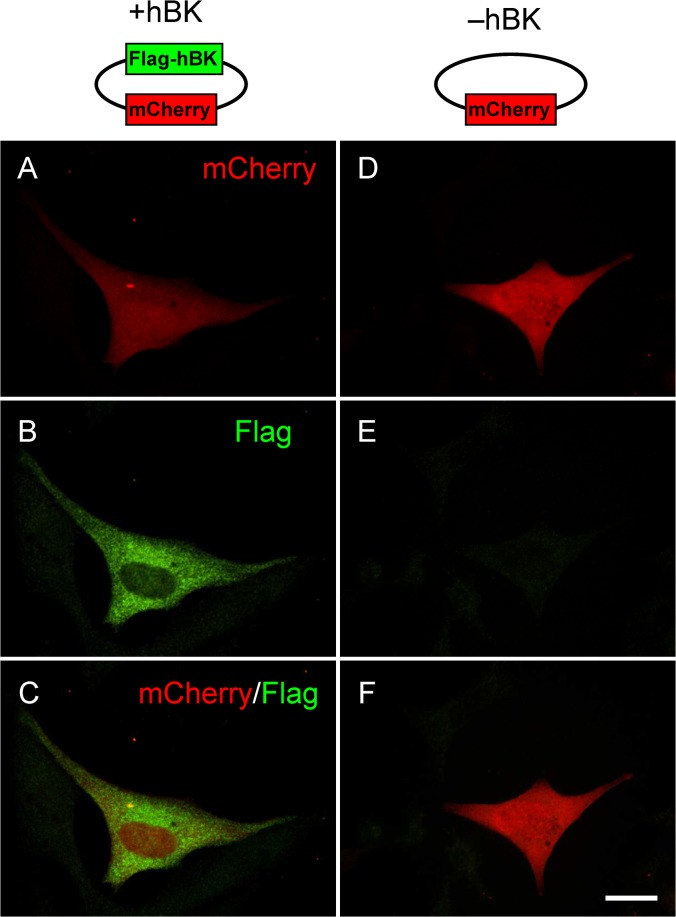
HL-1 Cell Immunofluorescence. Examples of HL-1 cells transfected with either +hBKα plasmid bicistronically expressing mCherry and Flag-tagged human BKα gene (top row) or Null plasmid with mCherry only (bottom row). **A, D)** mCherry (red) fluorescence; **B, E)** anti-Flag staining in green; **C, F)** merged images. Scale bar, 20 μm.

HL-1 cells were transfected for 48 to 72 hr with either +hBKα plasmid bicistronically expressing mCherry and Flag-tagged human BKα gene (Fig 1, top row) or Null plasmid with mCherry only (Fig 1, bottom row). Transfected cells identified by red fluorescence (Fig 1A and 1D) were used in all subsequent patch clamp experiments. As expected, +hBKα-transfected cells were positive for anti-Flag staining (Fig 1B and 1C) but not Null-transfected cells (Fig 1E and 1F).

**Fig 2:**
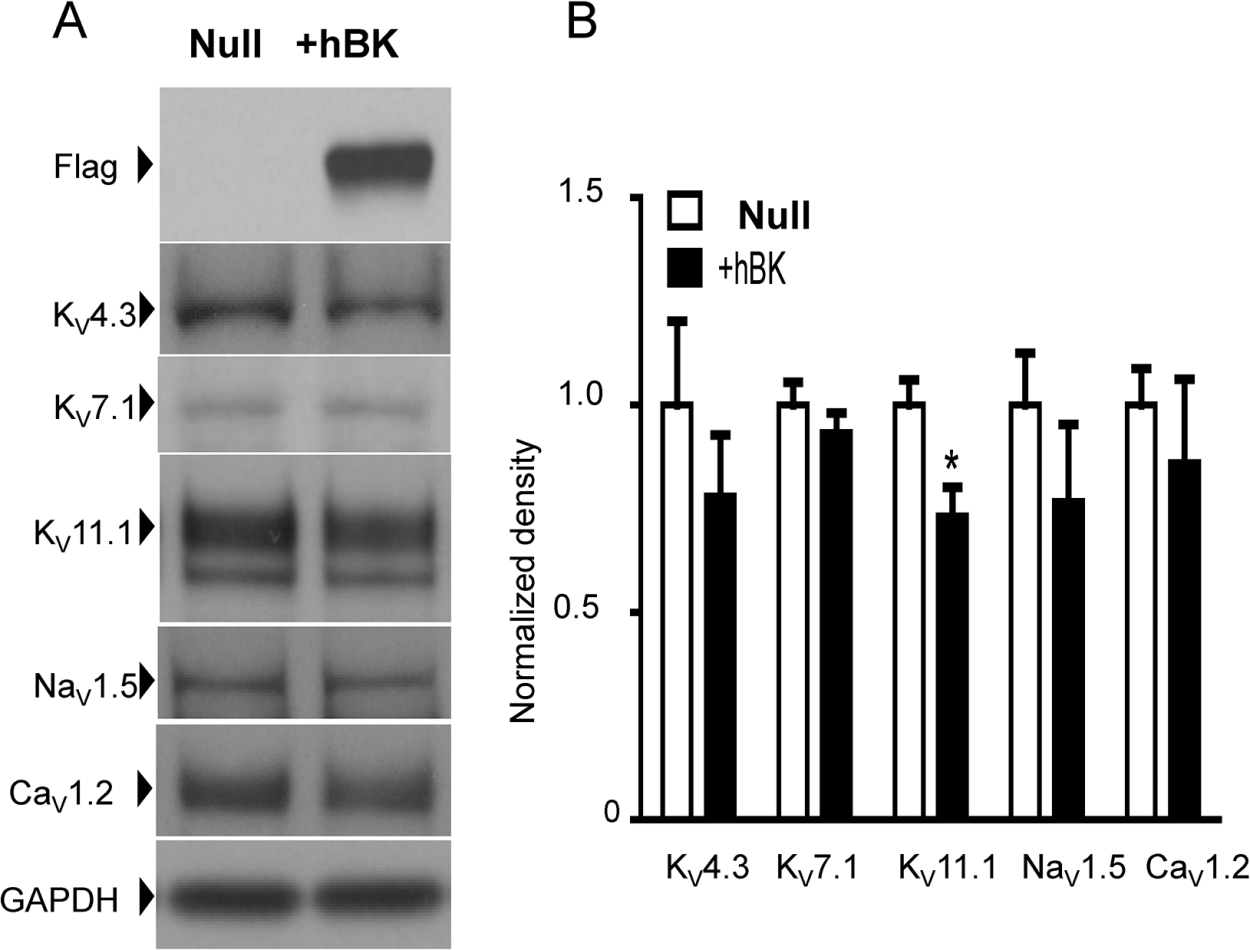
Characterization of ion channel proteins in HL-1 cells transfected with hBKα. **A)** Examples of Western blots comparing ion channel expression levels between HL-1 cells transfected with Null or +hBKα for 48 h. Pore-forming α-subunits of voltage-gated K^+^ channels (Kv4.3, Kv7.1, Kv11.1) native to HL-1 cells and voltage-gated Na^+^ (Na_v_1.5) and L-type Ca^2+^ (Ca_v_1.2) channels were probed. Molecular mass markers for each blot (in kD) are indicated on the right column. **B)** Density of immunoreactive bands were averaged from 5 separate HL-1 cell cultures transfected with Null or +hBKα (n = 5). * = significant difference from Null, P<0.05. See S1 Text and S1 Fig for complete Western blots of the data described in this Fig.

Changes in the expression of one ion channel type can alter the abundance of other ion channels, and this “remodeling” process includes cardiac myocytes that show a propensity to adapt their ion channel profiles [11,25]. For example, deletion of one or more K^+^ channel genes may affect the expression levels of other K^+^ channels in cardiac myocytes in vivo [26]. Also, changes in the expression level of K^+^ channels may alter the cell resting membrane potential, which subsequently can influence the abundance of L-type Ca^2+^ channels [27]. Based on these and other reports, we employed Western blotting to define the effect of +hBKα transfection on the expression of ion channels involved in generating APs in HL-1 cells. Our intent was to ensure that any shortening of APD attributed to hBKα expression was not related to an unintended increase of repolarizing K^+^ channels (KCND3, K_V_4.3; KCNQ1, K_V_7.1; HERG, K_V_11.1) or to a loss of depolarizing currents through voltage-gated Na^+^ channels (SCN5A, Na_V_1.5) or L-type Ca^2+^ channels (CACNA1C, Ca_V_1.2). First, we showed in Western blots using anti-Flag antibody that exogenous human BKα subunit was robustly expressed in +hBKα cells (Fig 2A). In the same protein preparation extracted from HL-1 cells expressing hBKα, we detected the voltage-gated K^+^ channels K_V_4.3, K_V_7.1 and K_V_11.1 that contribute to the repolarization phase of the AP (Fig 2A). Only K_v_11.1, detected as doublet immunoreactive bands at its predicted molecular weight of ~127 KD, showed a slight (~25%) but significant decrease in expression when either both bands or the single upper band were analyzed (Fig 2B). Notably, this loss of K_v_11.1 protein in +hBKα cells would be predicted to prolong APD, rather than contribute to shortening of APD after hBKα transfection as a confounding effect. Western blots did not reveal changes in expression of the pore-forming α subunits of the voltage-gated Na^+^ channel (Na_V_1.5), which mediates the upstroke of the AP in HL-1 cells, or of the L-type Ca^2+^ channel, (Ca_V_1.2) which is a critical contributor to the cardiac APD (Fig 2A and 2B).

### hBKα-transfected cells exhibit BK current

**Fig 3:**
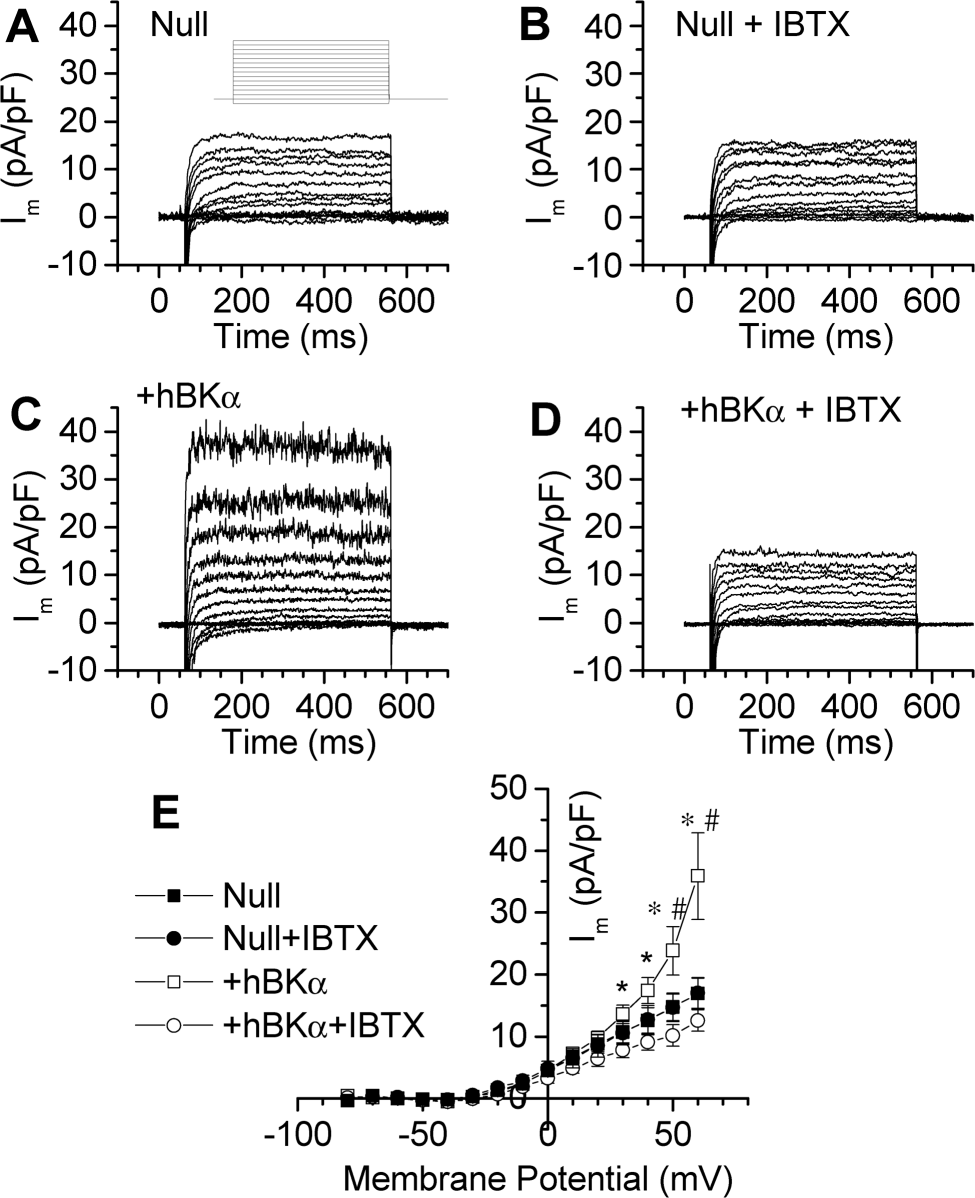
BK channel currents expressed in HL-1 cells. HL-1 cells were transfected with either a vector expressing only mCherry as a fluorescent marker (Null) or a plasmid containing mCherry and the human α-subunit of the large-conductance, Ca^2+^-activated K^+^ channel (+hBKα). **A)** Whole-cell currents recorded from a typical Null-transfected cell. Inset shows the protocol used in all studies. Cells were dialyzed with pipette solution containing 300 nM free Ca^2+^, clamped at -70 mV and pulsed to potentials between -80 and +60 mV in 10 mV steps for 500 ms. **B)** Currents recorded in the same cell as in panel A following application of 100 nM IBTX. **C)** Whole-cell currents recorded from a +hBKα-transfected cell. **D)** Currents recorded from the same cell as in panel C after application of 100 nM IBTX. **E)** Steady state I-V relationships for whole-cell current densities calculated from 5 to 8 cells for each condition. Currents recorded in Null cells (solid symbols) and +hBKα cells (open symbols) were recorded before and after application of 100 nM IBTX. +hBKα current densities were significantly different from current densities in IBTX at potentials positive to +20 mV. * = significant difference from IBTX measurements in +hBKα cells. # = significant difference between Null and +hBKα cells without IBTX.

HL-1 cells transfected with Null or +hBKα plasmids were subjected to the whole-cell voltage-clamp mode to record macroscopic membrane currents, and thereby identify functional BK channels. Unless otherwise noted, the predicted free-Ca^2+^ concentration in the pipette solution dialyzing the cells was buffered to 300 nM. This free-Ca^2+^ concentration is above normal resting levels in cardiac myocytes, but below levels of free-Ca^2+^ in contracting myocytes [28]; nanomolar free-Ca^2+^ concentrations are adequate to activate BK channels when present [21]. Fig 3 shows typical families of currents elicited from a holding potential of -70 mV with 500 ms pulses to potentials between -80 and +60 in 10 mV increments in Null (A) and +hBKα transfected HL-1 cells (C). The +hBKα-transfected cells showed a significant increase in outward current densities at membrane potentials positive to +20 mV compared to the Null-transfected cells, suggesting the expression of more functional K^+^ channels. The “noisy” outward currents at more positive potentials are typical of K^+^ currents mediated by BK channels, which mediate large single-channel amplitudes at positive voltages. To show that this increased outward current was mediated by BK channels, iberiotoxin (IBTX) a specific inhibitor of BK channels [29,30] was added to the patch-clamp chamber. Application of 100 nM IBTX did not significantly reduce the amplitude of outward currents in Null transfected cells (Fig 3B), but eliminated noisy outward currents in +hBKα cells (Fig 3D), confirming the presence of functional BK channels. Fig 3E shows average current-voltage (IV) relationships for all cells in this experiment. Outward current was significantly blocked by 100 nM IBTX at potentials above +20 mV in +hBKα cells (n = 8). Compared to Null cells (n = 5), values for the I-V relationship for +hBKα cells (n = 8) were significantly greater at potentials above +40 mV. However, in the presence of IBTX, I-V relationships were not significantly different between Null and +hBKα cells. These findings imply that the greater K^+^ current density in +hBKα cells relies on the presence of functional BK channels.

### +hBKα cells exhibit shortened APD

**Fig 4:**
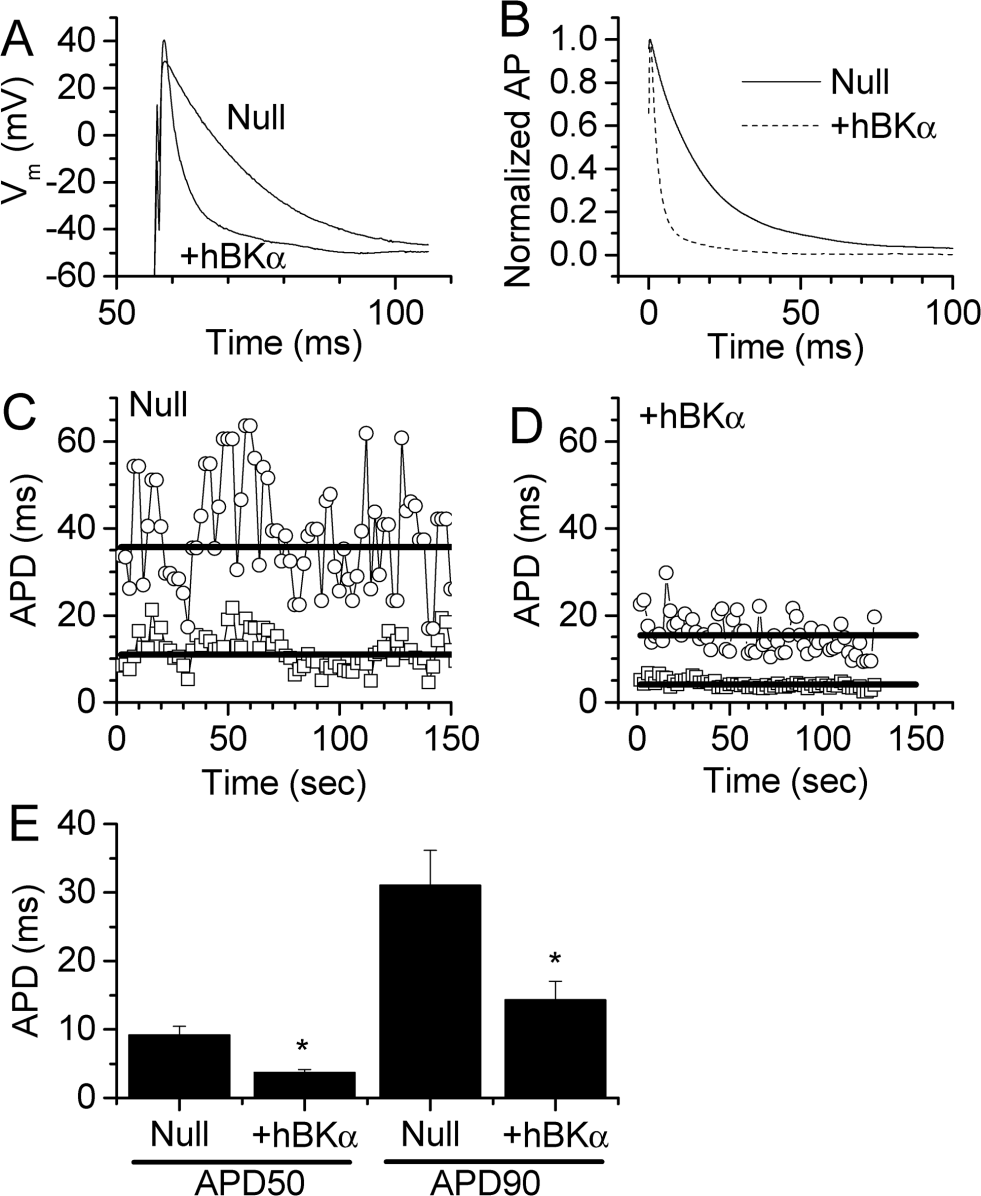
Effect of hBKα expression on APD. **A)** APs recorded from HL-1 cells stimulated at 0.5 Hz. The traces show a single AP evoked in a Null-transfected cell and hBKα-transfected cell dialyzed with pipette solution containing 300 nM free Ca^2+^. Anode break excitation was used to stimulate the cells. Thus, there is no baseline prior to the AP. Note the shorter APD in the hBKα- transfected cell compared to the Null cell. **B)** Average of 15–20 AP recorded from a single cell. **C)** A typical experiment in which APD values at 50% (APD50, open squares) and 90% (APD90, open circles) repolarization were measured every 2 sec in a Null cell. These measurements were then averaged to obtain the final APD value for each cell. Heavy solid line indicates calculated average APD50 (lower bar) and APD90 (upper) in this cell. **D)** APD measured in an +hBKα expressing HL-1 cell using the same protocol as in panel C. **E)** Summary of averaged APD values measured at 50% and 90% repolarization in HL-1 cells transfected with either Null or +hBKα plasmids. BK channel expression significantly reduced APD (n = 10–13). * = significant difference from Null measurements.

Individual HL-1 cardiac myocytes were patch clamped in the current-clamp mode at room temperature to compare APD values between Null and +hBKα cells. By stimulating the cells with anode break excitation, APs were elicited and recorded (Fig 4A). The resting membrane potential was highly variable from cell to cell (range, -40 to -75 mV) in the current-clamp mode. The reason for this variability was not readily apparent, but it did not appear to correlate with seal resistance or APD values (data not shown). To accommodate this variability, AP amplitude in each cell was normalized for maximum and minimum potential to compare APD values corresponding to 50% repolarization (APD50) and 90% repolarization (APD90) between cells. Normalized APs in a Null and +hBKα –transfected cell are plotted in Fig 4B. Sequential recordings of 15 to 20 APs were used to obtain average AP and APD values for each cell. Accordingly, Fig 4C shows measurements of APD every two seconds in the same Null-transfected cell as shown in Fig 4B, while Fig 4D shows a similar recording from the same +hBKα cell in Fig 4B. The solid bars show the average APD50 value (lower bar) and APD90 value (upper bar) in each cell. Fig 4E summarizes these results in experiments on 10 to 13 cells. In control nontransfected cells, AP durations of 8.3 ± 1.65 ms (APD50) and 25.2 ± 5.1 ms (APD90) (n = 8; data not shown) were comparable to HL-1 cells transfected with the Null plasmid (9.2 ± 1.2 ms (APD50) and 31.0 ± 5.1 ms APD90); n = 10). In contrast, HL-1 cells transfected with the +hBKα plasmid caused the APD to be significantly shorter (3.7 ± 0.5 ms (APD50) and 14.3 ± 2.8 ms APD90; n = 13). At both 50% and 90% repolarization, there was a significant reduction of APD in cells transfected with hBKα.

**Fig 5:**
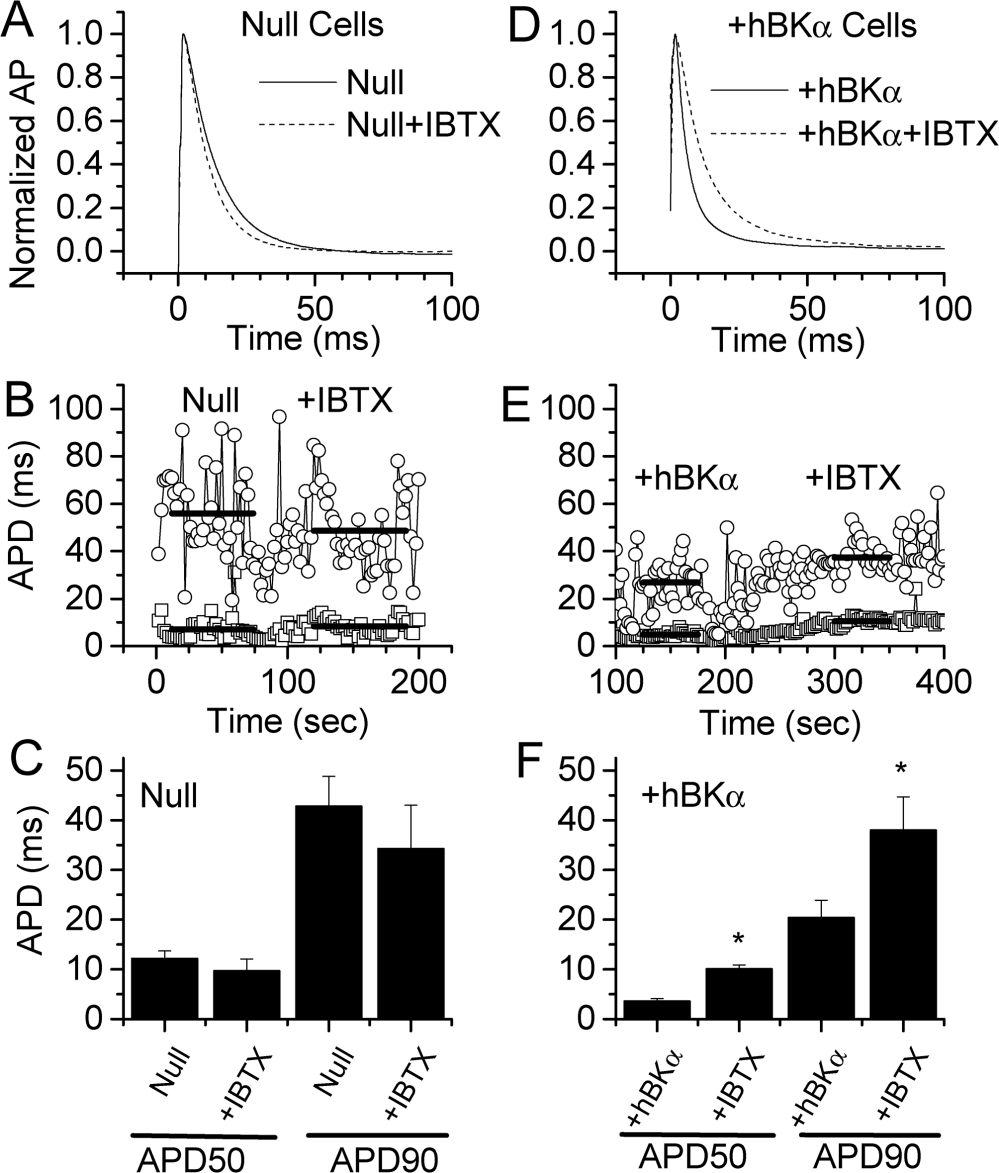
IBTX reverses the shortened APD in hBKα cells. Similar experiments to those in Fig 4 were performed on Null (A-C) and +hBKα (D-F) transfected cells, except APD50 and APD90 were calculated before and after addition of 100 nM IBTX. **A & D)** Averages of 15–20 APs recorded before and after exposure to 100 nM IBTX. **B & E)** Example of APD measured at 50% and 90% repolarization from AP stimulated at 0.5 Hz. IBTX was applied to these cells 90 (B) and 195 sec (E) after the start of recordings. Heavy solid bars indicate the time at which measurements were made to calculate the average APD50 (upper bars) and APD90 (lower bars). Placement of the bars shows the average value in these single cells. **C & F)** Average results from 5 cells each transfected with Null or +hBKα plasmids. IBTX had no effect on APD values in Null cells but reversed the APD shortening in +hBKα cells. * = significant difference from Null measurements.

To see if the shortened APD in hBKα-transfected cells was due to expression of functional BK channels, 100 nM IBTX was applied to both Null- and +hBKα-transfected cells. Using the same protocol as in Fig 4, Null (Fig 5A–5C) or +hBKα (Fig 5D–5F) transfected cells were stimulated at 0.5 Hz to elicit APs. Once stable APs were obtained, 100 nM IBTX was applied to determine the influence of BK channel-mediated K^+^ current on APD. As evident from the APs recorded, IBTX had no effect on APD in the Null-transfected cell (Fig 5A), but prolonged APD in the +hBKα-transfected cell (Fig 5D). In the same HL-1 cells, the recording of APD as a function of time is shown in Fig 5B and Fig 5E. While there is no apparent change in APD in the Null cell after IBTX was applied at 90 sec, there was a slow increase in APD the +hBKα cell following application of 100 nM IBTX at 195 seconds. The bars indicate the periods during which APD values were collected to obtain an average APD for each cell in control conditions and following IBTX application. Results are summarized in Fig 5C and Fig 5F for Null (n = 5) and +hBKα (n = 5) cells. Null cells showed no significant change in APD at either 50% (12.2 ± 1.5 ms control; 9.7 ± 2.4 ms IBTX) or 90% repolarization (42.8 ± 5.9 ms control; 34.3 ± 8.7 ms IBTX). However, +hBKα-transfected cells had a shorter APD consistent with the results in Fig 4E. This shortened APD in +hBKα cells was significantly prolonged by IBTX at both 50% (3.6 ± 0.5 ms control; 10.1 ± 0.8 ms IBTX) and 90% repolarization (20.4 ± 5.5 ms control; 37.9 ± 6.8 ms IBTX). After IBTX, APD50 and APD90 values for +hBKα cells were not significantly different from corresponding values in Null cells.

### Shortened AP in +hBKα cells is absent with low free Ca^2+^

**Fig 6:**
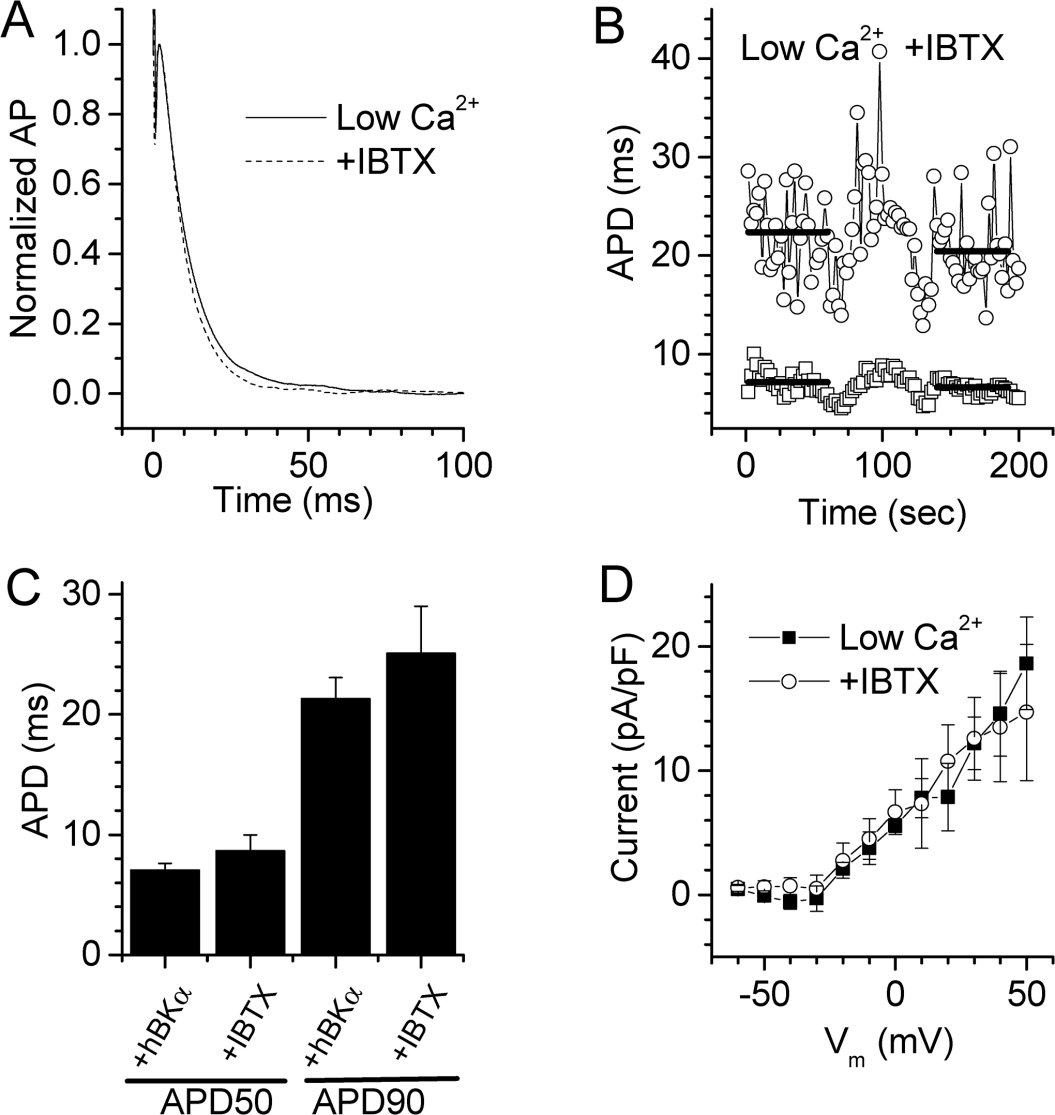
Lowering free Ca^2+^ prevents hBKα-induced shortening of APD and eliminates BK current. **A)** Averaged AP recorded in a +hBKα cell dialyzed with pipette solution containing low (10 nM) free Ca^2+^ before and after application of 100 nM IBTX. **B)** Plot of APD50 and APD90 measured in the same +hBKα cell as in A. APs were stimulated at 0.5 Hz, and IBTX was applied to the cell at 75 sec after the start of the recording. Heavy solid bars indicate the time at which measurements were made to calculate the average APD50 (lower bars) and APD90 (upper bars). The bar placement shows the average values in this single cell. **C)** APD measured before and after application of 100 nM IBTX in five HL-1 cells transfected with +hBKα and dialyzed with 10 nM free Ca^2+^. IBTX did not significantly increase APD50 or APD90 in these cells. **D)** Steady state I-V relationships of whole-cell current densities calculated from +hBKα-transfected cells dialyzed with 10 nM free Ca^2+^ before (solid symbol) and after (open symbol) application of 100 nM IBTX. These cells exhibited no IBTX-sensitive current (n = 5). Cells were clamped at -70 mV and pulsed to potentials between -80 and +60 mV in 10 mV steps for 500 ms (see protocol inset to Fig 3A).

To confirm that the BK channel-mediated repolarizing current was responsible for the shortened APD in +hBKα, we lowered the free Ca^2+^ in the pipette solution from 300 nM to 10 nM to minimize activation of BK channels. Fig 6A shows APs recorded from a +hBKα- transfected cell before and after application of 100 nM IBTX. Unlike +hBK cells dialyzed with 300 nM Ca^2+^, in which IBTX restored shortened APD values to those of Null cells (Fig 5D–5F), IBTX failed to lengthen APD in this +hBKα cell dialyzed with 10 nM free Ca^2+^ (Fig 6A). This finding implied that the low free Ca^2+^ prevented activation of the BK channels expressed in +hBKα cells, and eliminated an effect of BK channel block by IBTX on APD. Fig 6B shows the continual recording of APD in this same cell as a function of time and shows that IBTX added at 60 sec did not increase APD50 (upper bar) or APD90 (lower bar). Fig 6C summarizes similar APD data obtained from five +hBKα cells dialyzed with 10 nM free Ca^2+^. IBTX had no significant effect on APD at either 50% (7.0 ± 0.5 ms control; 8.6 ± 1.3 ms IBTX) or 90% repolarization (21.3 ± 1.8 ms control; 25.1 ± 3.9 ms IBTX). In a final set of studies, we recorded whole-cell currents in voltage-clamped +hBKα cells dialyzed with 10 nM free Ca^2+^, and verified the absence of IBTX-sensitive current (Fig 6D; compare with Fig 3E). These findings suggest that the free-Ca^2+^ concentration in HL-1 cardiomyocytes regulates the activity of BK channels, and as a result, their ability to influence APD. The finding that lowering the free-Ca^2+^ concentration in the cell dialysate prevented APD shortening in +hBKα –transfected cells further implies that K^+^ efflux through BK channels was responsible for this effect in HL-1 cells.

## Discussion

Our study provides initial evidence that expression of the pore-forming BKα-subunit in HL-1 cardiac cells results in a population of functional K^+^ channels capable of shortening APD. The shortening of APD in +hBKα-transfected cells was reversed by the BK channel inhibitor, IBTX, and by lowering intracellular free Ca^2+^, which is expected to shift the voltage-dependent activation of BK channels to more depolarized potentials [21]. In contrast, Null-transfected cells lacking IBTX-sensitive BK channels exhibited longer APD values comparable to those of untransfected HL-1 cells. These findings suggest that the shortening effect of +hBKα transfection on APD was, indeed, due to a novel repolarizing K^+^ current mediated by exogenous BK channels. We also failed to detect endogenous expression of BK channel protein in HL-1 cells, a finding also reported for other cardiac preparations [15,16].

Regardless of these encouraging findings, we considered the possibility that +hBKα transfection disrupted the expression of native channels in HL-1 cells involved in AP repolarization. Alteration of electrophysiological cues including changes in membrane potential can influence ion channel expression in multiple cell types, including cardiac myocytes [11,25–27,31]. However, our Western blot screening of the most prevalent ion channels in HL-1 cells revealed that only the abundance of K_V_11.1 (ERG) was different between Null- and +hBKα-transfected cells. The expression of this repolarizing K^+^ channel was about 25% less in +hBKα cells. As Kv11.1 is a component of I_Kr_, which contributes to AP repolarization in HL-1 cells [9], it is possible that a reduction in its expression could increase APD. However, this outcome was not observed in +hBKα expressing cells, which exhibited a 53% reduction in APD compared to Null cells. These findings strengthened the conclusion that the shortening of APD measured in +hBKα -expressing cells resulted from the repolarizing influence of exogenous human BK channels and was not caused by an off-target effect.

### Choice of the BK channel as a repolarizing intervention

For this study, we chose to investigate the BK channel as a potential repolarizing therapeutic based on several of its unique properties. First, BKα subunits are encoded by a single gene, which simplifies plasmid design and ensures that +hBKα transfection results in a predictable pore-forming structure. Second, BK channels are uniquely activated by membrane depolarization and intracellular free Ca^2+^, suggesting these stimuli during the cardiac AP should effectively open exogenous BK channels to enhance the rate of repolarization. Indeed, when the predicted free-Ca^2+^ concentration in the pipette solution dialyzing HL-1 cells was buffered to 300 nM, which represents a free-Ca^2+^ concentration higher than resting levels in cardiac myocytes but below levels of peak Ca^2+^ measured in contracting myocytes [28], it was adequate to activate BK channels during depolarizing steps in the voltage-clamp mode, or during elicited AP in current-clamp recordings [21]. In contrast, a low sub-physiological free-Ca^2+^ concentration (10 nM) prevented activation of expressed hBK channels. Third, in contrast to relatively small single-channel conductances ranging from 9 to 40 pS for K^+^ channel types including the slow, rapid and ultra-rapid delayed rectifiers (I_Ks_, I_Kr_, I_Kur_), inward rectifier (I_K1_) and ATP dependent (I_K-ATP_) native to cardiac myocytes [32], BK channels exhibit a “big” single-channel conductance of 100 to 300 pS [20]. The large unitary current amplitudes mediated by BK channels infers that relatively few channel openings can generate sufficient K^+^ current to induce membrane repolarization.

Surprisingly, although BK channels appear to be ubiquitously expressed in most cell types, most studies have reported an absence of BK channel protein in cardiac sarcolemmal preparations [15,16]. Similarly, we failed to detect native BK channels in the HL-1 cardiac cell line used in this study. However, Imlach et al. [33] recently reported that the BK channel inhibitors paxilline and lolitrem B induce bradycardia in conscious wild-type mice and in isolated perfused hearts of these animals. The bradycardia induced by BK channel inhibitors was absent in hearts of BKα knock-out mice. The authors speculate that BK channels in the sino-atrial node may regulate firing frequency, or alternatively, if BK channels exist in mitochondria of sinus nodal cells, they might influence heart rate through metabolic mechanisms. Indeed, the presence of BK channels in the inner layer of the cardiac mitochondrial membrane has been demonstrated in mouse cardiac myocytes [15] and these mitochondrial channels apparently can mediate K^+^ removal from the cytosol to protect against myocardial ischemia [16]. Thus, although BK channels are not expressed in the sarcolemma of native cardiac myocytes, they may regulate cardiac function by an alternative unique mechanism.

### Limitations of the study

We used the HL-1 cell model in this study because it displays a combination of important assets. HL-1 cells are a stable expression model, exhibit ease of transfection unlike primary cardiac myocytes, are amenable to patch-clamp and current-clamp studies, and express a complement of ion channels on the cell surface including voltage gated Na^+^, Ca^2+^ and K^+^ channels that resemble the ion channel types found in primary cultures of mammalian cardiac cells [7,9,13,24,34,35]. Nonetheless, we acknowledge that there are significant limitations related to our choice of the HL-1 cell line. First, HL-1 cells are derived from an atrial myocyte tumor, and they lack features of native cardiac myocytes that include lack of structural regularity, embryonic phenotype of some genes and they continually divide in culture. Most importantly for the present study, HL-1 cells have a much shorter AP than adult mammalian ventricular myocytes including those of humans. As a result of this difference in AP phenotype, the depolarizing stimulus and rise in intracellular free Ca^2+^ associated with AP generation (presumed to activate BK channels), will likely be different between HL-1 cells and human ventricular myocytes. An additional limitation is the small size of HL-1 cells, which showed average cell membrane capacitance values of ~15 to 30 pF in this study. In contrast, single human ventricular myocytes exhibit cell capacitances of ~100–200 pF [36], suggesting that a higher abundance of BK channels may be required to exert a powerful repolarizing current in these cells. Finally, it is well recognized that the physiological and pharmacological properties of BK channels can be profoundly influenced by the presence of ancillary proteins and intracellular signaling molecules. For example, accessory β-subunits arising from four different genes (BKβ1–4) can bind in 1:1 stoichiometry with BKα subunits to drastically alter BK channel phenotype [21]. For example, co-assembly of BKα pore-forming proteins with BKβ1 subunits increases the Ca^2+^-sensitivity of BK channels [21] to promote repolarizing K^+^ current, whereas BKβ4 subunits reduce surface expression of BKα proteins to lower BK channel abundance, and also confer resistance to the inhibitory effect of iberiotoxin [37]. The complexities of BK channel regulation, which are tissue-specific and only partially resolved [38], imply that the proof-of-principle studies reported here serve only as a foundation for more detailed studies needed to evaluate the benefit of exogenous BK channel expression in shortening APD in more physiological cardiac myocyte preparations.

### Relevance of findings to LQTS

Our findings raise the possibility of using the hBKα gene as a potential therapeutic for genetic forms of LQTS. About 80 to 90% of LQTS cases are associated with loss of function of two K^+^ channels, the slow and rapid delayed rectifier K^+^ channels [4]. Loss of function of either of these K^+^ channels leads to a prolonged APD, which subsequently results in a net depolarization of the ventricular myocytes. This depolarization culminates in a rise in intracellular free Ca^2+^ mediated by activation of voltage-gated Ca^2+^ channels and less efflux through Na^+^/Ca^2+^ exchange, conditions that promote K^+^ efflux through BK channels. This mechanism underlies our rationale for identifying expression of exogenous BK channels as a potential therapeutic intervention for LQTS.

## Supporting Information

S1 Fig Original scans of Western blots from control (C), Null (N) and hBK (B)-transfected HL-1 cells. Arrowheads mark the expected band size of each protein.
(TIFF)

S1 Text Supplement to Stimers et al. (DOCX)

## References

[1] Schwartz PJ, Crotti L, Insolia R (2012) Long-QT syndrome: from genetics to management. Circ Arrhythm Electrophysiol 5: 868–877. 5/4/868 [pii]; 10.1161/CIRCEP.111.962019 22895603 PMC3461497

[2] Schwartz PJ, Stramba-Badiale M, Crotti L, Pedrazzini M, Besana A, Bosi G, et al (2009) Prevalence of the congenital long-QT syndrome. Circulation 120: 1761–1767. CIRCULATIONAHA.109.863209 [pii]; 10.1161/CIRCULATIONAHA.109.863209 19841298 PMC2784143

[3] Hedley PL, Jorgensen P, Schlamowitz S, Wangari R, Moolman-Smook J, Brink PA, et al (2009) The genetic basis of long QT and short QT syndromes: a mutation update. Hum Mutat 30: 1486–1511. 10.1002/humu.21106 19862833

[4] Szeliga MA, Hedley PL, Green CP, Moller DV, Christiansen M (2010) Long QT syndrome—a genetic cardiac channelopathy. Kardiol Pol 68: 575–583. 20491026

[5] Schwartz PJ, Spazzolini C, Priori SG, Crotti L, Vicentini A, Landolina M, et al (2010) Who are the long-QT syndrome patients who receive an implantable cardioverter-defibrillator and what happens to them?: data from the European Long-QT Syndrome Implantable Cardioverter-Defibrillator (LQTS ICD) Registry. Circulation 122: 1272–1282. CIRCULATIONAHA.110.950147 [pii]; 10.1161/CIRCULATIONAHA.110.950147 20837891

[6] So EC, Hsing CH, Liang CH, Wu SN (2012) The actions of mdivi-1, an inhibitor of mitochondrial fission, on rapidly activating delayed-rectifier K(+) current and membrane potential in HL-1 murine atrial cardiomyocytes. Eur J Pharmacol 683: 1–9. S0014-2999(12)00148-3 [pii]; 10.1016/j.ejphar.2012.02.012 22374256

[7] Claycomb WC, Lanson NA Jr., Stallworth BS, Egeland DB, Delcarpio JB, Bahinski A, et al (1998) HL-1 cells: A cardiac muscle cell line that contracts and retains phenotypic characteristics of the adult cardiomyocyte. Proc Natl Acad Sci USA 95: 2979–2984. 9501201 10.1073/pnas.95.6.2979 PMC19680

[8] McWhinney CD, Hansen C, Robishaw JD (2000) Alpha-1 adrenergic signaling in a cardiac murine atrial myocyte (HL-1) cell line. Mol Cell Biochem 214: 111–119. 11195782 10.1023/a:1007129723949

[9] Toyoda F, Ding WG, Zankov DP, Omatsu-Kanbe M, Isono T, Horie M, et al (2010) Characterization of the rapidly activating delayed rectifier potassium current, I (Kr), in HL-1 mouse atrial myocytes. J Membr Biol 235: 73–87. 10.1007/s00232-010-9257-2 20490473

[10] Nissen JD, Diness JG, Diness TG, Hansen RS, Grunnet M, Jespersen T (2009) Pharmacologically induced long QT type 2 can be rescued by activation of IKs with benzodiazepine R-L3 in isolated guinea pig cardiomyocytes. J Cardiovasc Pharmacol 54: 169–177. 10.1097/FJC.0b013e3181af6db3 19568177

[11] Schmitt N, Grunnet M, Olesen SP (2014) Cardiac potassium channel subtypes: new roles in repolarization and arrhythmia. Physiol Rev 94: 609–653. 10.1152/physrev.00022.2013 24692356

[12] Yang T, Snyders D, Roden DM (2001) Drug block of I(kr): model systems and relevance to human arrhythmias. J Cardiovasc Pharmacol 38: 737–744. 11602820 10.1097/00005344-200111000-00010

[13] Stimers JR, Ha J, Hastings SL (2002) Electrophysiological characterization of mouse atrial HL-1 cells. Biophys J 82: 95a.

[14] Mikuni I, Torres CG, Bakshi T, Tampo A, Carlson BE, Bienengraeber MW, Kwok WM (2015) Enhanced Effects of Isoflurane on the Long QT Syndrome 1-associated A341V Mutant. Anesthesiol. 10.1097/ALN.0000000000000583 PMC4366337 25585005

[15] Ko JH, Ibrahim MA, Park WS, Ko EA, Kim N, Warda M, et al (2009) Cloning of large-conductance Ca(2+)-activated K(+) channel alpha-subunits in mouse cardiomyocytes. Biochem Biophys Res Commun 389: 74–79. S0006-291X(09)01667-2 [pii]; 10.1016/j.bbrc.2009.08.087 19699717

[16] Xu W, Liu Y, Wang S, McDonald T, Van Eyk JE, Sidor A, et al (2002) Cytoprotective role of Ca2+- activated K+ channels in the cardiac inner mitochondrial membrane. Science 298: 1029–1033. 10.1126/science.1074360 298/5595/1029 [pii]. 12411707

[17] Li W, Gao SB, Lv CX, Wu Y, Guo ZH, Ding JP, et al (2007) Characterization of voltage-and Ca2+-activated K+ channels in rat dorsal root ganglion neurons. J Cell Physiol 212: 348–357. 10.1002/jcp.21007 17523149

[18] Hu XQ, Zhang L (2012) Function and regulation of large conductance Ca(2+)-activated K+ channel in vascular smooth muscle cells. Drug Discov Today 17: 974–987. S1359-6446(12)00125-0 [pii]; 10.1016/j.drudis.2012.04.002 22521666 PMC3414640

[19] Sun X, Zaydman MA, Cui J (2012) Regulation of Voltage-Activated K(+) Channel Gating by Transmembrane beta Subunits. Front Pharmacol 3: 63 10.3389/fphar.2012.00063 22529812 PMC3328208

[20] Yang H, Zhang G, Cui J (2015) BK channels: multiple sensors, one activation gate. Front Physiol 6: 29 10.3389/fphys.2015.00029 25705194 PMC4319557

[21] Brenner R, Jegla TJ, Wickenden A, Liu Y, Aldrich RW (2000) Cloning and functional characterization of novel large conductance calcium-activated potassium channel beta subunits, hKCNMB3 and hKCNMB4. J Biol Chem 275: 6453–6461. 10692449 10.1074/jbc.275.9.6453

[22] Rhee SW, Starr T, Forsten-Williams K, Storrie B (2005) The steady-state distribution of glycosyltransferases between the Golgi apparatus and the endoplasmic reticulum is approximately 90:10. Traffic 6: 978–990. TRA333 [pii]; 10.1111/j.1600-0854.2005.00333.x 16190979

[23] Hamill OP, Marty A, Neher E, Sakmann B, Sigworth FJ (1981) Improved patch-clamp techniques for high resolution current recording from cells and cell free membrane patches. Pflugers Arch 391: 85–100. 6270629 10.1007/BF00656997

[24] Telemaque S, Sonkusare S, Grain T, Rhee SW, Stimers JR, Rusch NJ, et al (2008) Design of mutant beta2 subunits as decoy molecules to reduce the expression of functional Ca2+ channels in cardiac cells. J Pharmacol Exp Ther 325: 37–46. jpet.107.128215 [pii]; 10.1124/jpet.107.128215 18184831 PMC4939485

[25] Michael G, Xiao L, Qi XY, Dobrev D, Nattel S\par (2009) Remodelling of cardiac repolarization: how homeostatic responses can lead to arrhythmogenesis. Cardiovasc Res 81: 491–499. 10.1093/cvr/cvn266 18826964

[26] Guo W, Li H, London B, Nerbonne JM (2000) Functional consequences of elimination of i(to,f) and i(to,s): early afterdepolarizations, atrioventricular block, and ventricular arrhythmias in mice lacking Kv1.4 and expressing a dominant-negative Kv4 alpha subunit. Circ Res 87: 73–79. 10884375 10.1161/01.res.87.1.73

[27] Pesic A, Madden JA, Pesic M, Rusch NJ (2004) High blood pressure upregulates arterial L-type Ca2+ channels: is membrane depolarization the signal? Circ Res 94: e97–104. 10.1161/01.RES.0000131495.93500.3c 01.RES.0000131495.93500.3c [pii]. 15131006

[28] Hohendanner F, McCulloch AD, Blatter LA, Michailova AP (2014) Calcium and IP3 dynamics in cardiac myocytes: experimental and computational perspectives and approaches. Front Pharmacol 5: 35 10.3389/fphar.2014.00035 24639654 PMC3944219

[29] Liu Y, Savtchouk I, Acharjee S, Liu SJ (2011) Inhibition of Ca2+-activated large-conductance K+ channel activity alters synaptic AMPA receptor phenotype in mouse cerebellar stellate cells. J Neurophysiol 106: 144–152. jn.01107.2010 [pii]; 10.1152/jn.01107.2010 21562198 PMC3129717

[30] Candia S, Garcia ML, Latorre R (1992) Mode of action of iberiotoxin, a potent blocker of the large conductance Ca(2+)-activated K+ channel. Biophys J 63: 583–590. S0006-3495(92)81630-2 [pii]; 10.1016/S0006-3495(92)81630-2 1384740 PMC1262182

[31] Telemaque S, Marsh JD (2009) Modification of cardiovascular ion channels by gene therapy. Expert Rev Cardiovasc Ther 7: 939–953. 10.1586/erc.09.76 19673672

[32] Tamargo J, Caballero R, Gomez R, Valenzuela C, Delpon E (2004) Pharmacology of cardiac potassium channels. Cardiovasc Res 62: 9–33. 10.1016/j.cardiores.2003.12.026 S0008636304000021 [pii]. 15023549

[33] Imlach WL, Finch SC, Miller JH, Meredith AL, Dalziel JE (2010) A role for BK channels in heart rate regulation in rodents. PLoS One 5: e8698 10.1371/journal.pone.0008698 20090847 PMC2806827

[34] Wondergem R, Graves BM, Li C, Williams DL (2012) Lipopolysaccharide prolongs action potential duration in HL-1 mouse cardiomyocytes. Am J Physiol Cell Physiol 303: C825–C833. ajpcell.00173.2012 [pii]; 10.1152/ajpcell.00173.2012 22895260 PMC3469715

[35] Yang T, Kupershmidt S, Roden DM (1995) Anti-minK antisense decreases the amplitude of the rapidly activating cardiac delayed rectifier K^+^ current. Circ Res 77: 1246–1253. 7586238 10.1161/01.res.77.6.1246

[36] Polak S, Fijorek K (2012) Inter-individual variability in the pre-clinical drug cardiotoxic safety assessment—analysis of the age-cardiomyocytes electric capacitance dependence. J Cardiovasc Transl Res 5: 321–332. 10.1007/s12265-012-9357-8 22411323 PMC3349867

[37] Shruti S, Urban-Ciecko J, Fitzpatrick JA, Brenner R, Bruchez MP, Barth AL (2012) The brain-specific Beta4 subunit downregulates BK channel cell surface expression. PLoS One 7: e33429 10.1371/journal.pone.0033429 PONE-D-11-22216 [pii]. 22438928 PMC3306404

[38] Contreras GF, Castillo K, Enrique N, Carrasquel-Ursulaez W, Castillo JP, Milesi V, et al (2013) A BK (Slo1) channel journey from molecule to physiology. Channels (Austin) 7: 442–458. 26242 [pii]; 10.4161/chan.26242 24025517 PMC4042479

