# Supplementary figures and images for: Overexpression of the Large-Conductance, Ca2+-Activated K+ (BK) Channel Shortens Action Potential Duration in HL-1 Cardiomyocytes
Source: PLoS One. 2015 Jun 19;10(6):e0130588. doi: 10.1371/journal.pone.0130588 (PMC4474436; doi:10.1371/journal.pone.0130588)

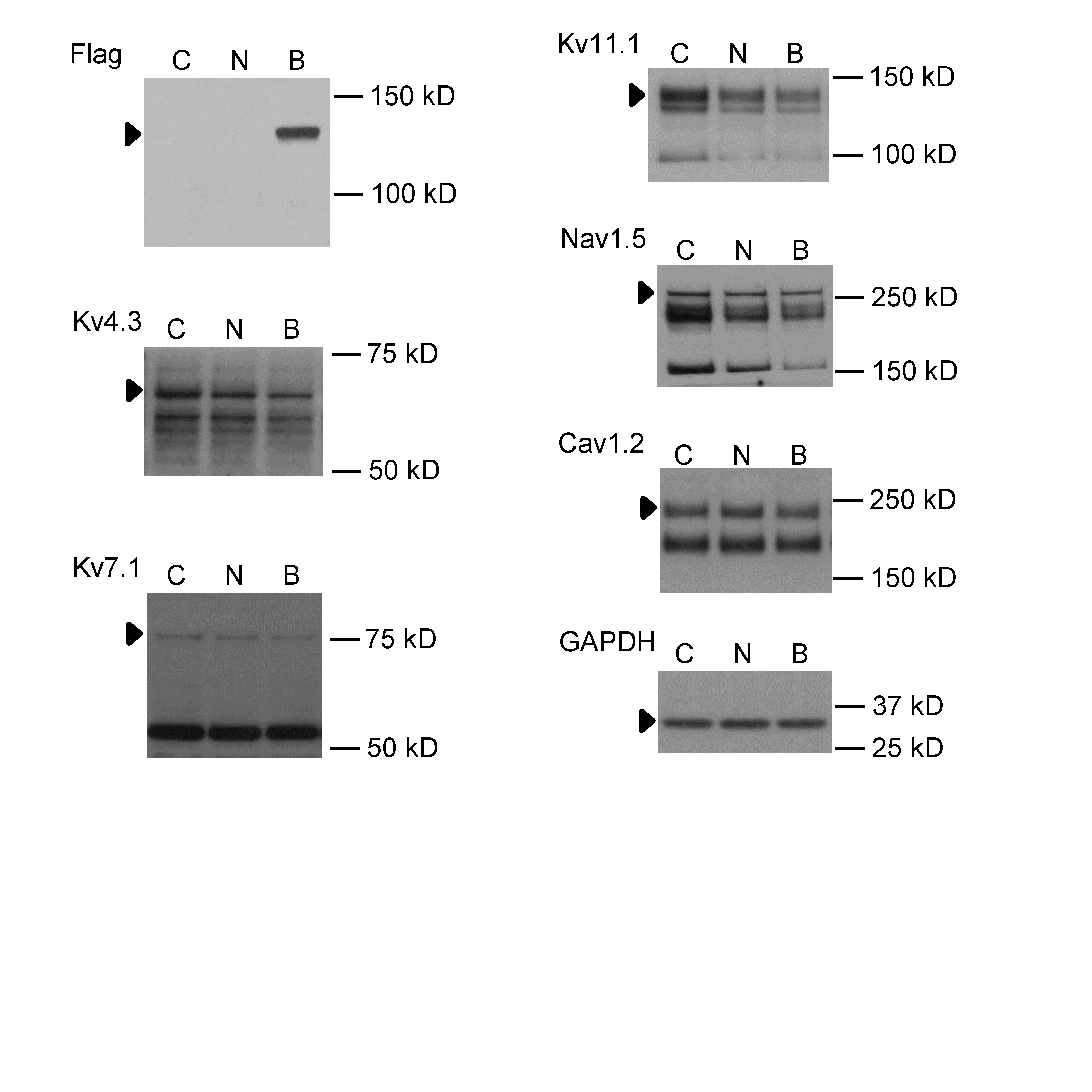

Supplement: S1 Fig — Arrowheads mark the expected band size of each protein. (TIFF) [file pone.0130588.s001.tiff]
