# Supplementary material for: Overexpression of the Large-Conductance, Ca2+-Activated K+ (BK) Channel Shortens Action Potential Duration in HL-1 Cardiomyocytes
Source: PLoS One. 2015 Jun 19;10(6):e0130588. doi: 10.1371/journal.pone.0130588 (PMC4474436; doi:10.1371/journal.pone.0130588)
Supplement: S1 Text — (DOCX) [file pone.0130588.s002.docx]

Supplement to Stimers et al.

The supplemental figure S1_Fig. shows complete information about the Western blots made to analyze the expression of various ion channels in HL-1 cells. The figure shows complete Western blots for each ion channel comparing Control, Null-transfected and +hBKα-transfected HL-1 cells in this study. These images of Western blots have not been digitally edited in any way. Further details of this study are presented in the associated manuscript.
